# Artificial intelligence-based model for the interpretation and reporting of standard automated perimetry

**DOI:** 10.5935/0004-2749.2024-0270

**Published:** 2025-06-24

**Authors:** Joacy Pedro Franco David, Alexandre Antonio Marques Rosa, Rafael Scherer, Cláudio Eduardo Corrêa Teixeira, Douglas Costa

**Affiliations:** 1 Universidade Federal do Pará, Belém, PA, Brazil; 2 Bascom Palmer Eye Institute, Miami, Miami, FL, United States of America; 3 Centro Universitário Estado do Pará, Belém, PA, Brazil

**Keywords:** Glaucoma, Disease progression, Perimetry, Visual Fields, Visual field tests, Artificial intelligence, Neural networks, computers, Machine learning

## Abstract

**Purpose:**

Standard automated perimetry has been the standard method for measuring visual field changes for several years. It can measure an individual’s ability to detect a light stimulus from a uniformly illuminated background. In the management of glaucoma, the primary objective of perimetry is the identification and quantification of visual field abnormalities. It also serves as a longitudinal evaluation for the detection of disease progression. The development of artificial intelligence--based models capable of interpreting tests could combine technological development with improved access to healthcare.

**Methods:**

In this observational, cross-sectional, descriptive study, we used an artificial intelligence-based model [Inception V3] to interpret gray-scale crops from standard automated perimetry that were performed in an ophthalmology clinic in the Brazilian Amazon rainforest between January 2018 and December 2022.

**Results:**

The study included 1,519 standard automated perimetry test results that were performed using Humphrey HFA-II-i-750 (Zeiss Meditech). The Subsequently, 70%, 10%, and 20% of the dataset were used for training, validation, and testing, respectively. The model achieved 80% (68.23%-88.9%) sensitivity and 94.64% (88.8%-98%) specificity for detecting altered perimetry results. Furthermore, the area under the receiver operating characteristic curve was 0.93.

**Conclusions:**

The integration of artificial intelligence in the diagnosis, screening, and monitoring of pathologies represents a paradigm shift in ophthalmology, enabling significant improvements in safety, efficiency, availability, and accessibility of treatment.

## INTRODUCTION

Standard automated perimetry (SAP) has been the standard method for evaluating visual field changes for several years. SAP measures an individual’s ability to detect a light stimulus emitted from a uniformly illuminated background^(1)^. The main goals of SAP in glaucoma management are the identification and quantification of abnormalities in the visual field and serving as a longitudinal assessment method for the detection of disease progression^(2)^.

In SAP, the patient’s central and peripheral vision sensitivity is quantified using algorithms that precisely determine the sensitivity threshold at each visual field location. At these locations, the light stimulus is presented at different intensities, and the patient must respond whenever they perceive the applied stimulus. Subsequently, a probability analysis is performed by comparing these responses with those of healthy individuals and individuals with the disease that are included in a database^(2)^.

Artificial intelligence (AI) is a branch of computer science that attempts to emulate human behavior or reasoning in computers. AI broadly encompasses several modalities, including machine learning (ML) and deep learning (DL)^(3)^. It was first discussed in 1956^(4)^ and was defined as a technology capable of imitating human behavior. Since then, there have been significant developments in AI and the creation of subfields such as ML and DL.

The World Economic Forum considers AI as the fourth industrial revolution in the history of humankind. AI modalities such as ML and DL have been widely used in the medical field and are generally applied in the processing of medical imaging. They have exhibited promising results in diagnosing various pathologies such as tuberculosis via radiographic examination, cutaneous melanoma via photographs, and lymph node metastasis secondary to breast cancer on the basis of histopathological examination^(5^-^7)^.

In ophthalmology, AI and DL have been used to ana-lyze ophthalmological images, especially optical coherence tomography (OCT) and color fundus photography. Furthermore, AI has demonstrated promising results in the interpretation of SAP, which is widely used in the diagnosis and monitoring of glaucoma^(8^,^9)^.

In this study, we aimed to develop an AI program capable of differentiating normal SAP results from pathological SAP results and categorizing them as normal and altered results, respectively. Furthermore, we aimed to evaluate the performance of the AI model and demonstrate its effectiveness as a screening tool for visual field pathologies.

## METHODS

This is a cross-sectional and descriptive study of the creation of an AI model capable of differentiating normal SAP records from pathological SAP records. At this time, the model does not aim to determine the pathology or classify the severity of a specific disease as its purpose is to serve as a modality for screening SAP records.

### Data collection

The study was developed using a database that included the anonymous results of SAPs that had been performed in patients treated at two private practices in Belém do Pará between January 2018 and December 2022. The research sample consisted of 1,519 SAP records, which did not state the specific baseline pathologies, visual status, race, age, or sex. All exams were performed using a Humphrey HFA II-i 750i^®^ perimeter (Carl Zeiss Meditec, Inc). All the SAPs were based on the Swedish interactive threshold algorithm, which is a rapid method of examination

### Statistical analysis

After data collection, the SAP reports were analyzed by two glaucoma specialists and classified as normal or altered (visual field defects). Classical glaucoma changes such as arcuate defects, nasal steps, and paracentral scotomas and visual field defects emerging from other pathologies such as bitemporal hemianopsias secondary to central nervous system tumors were considered as altered visual fields.

The developed AI model utilized InceptionV3, a convolutional neural network (CNN), for transfer learning from a pre-trained model. Specifically, the model used the printout gray-map images generated by the SAP test, leveraging InceptionV3’s capability to effectively learn discriminative features. The deeper layers of the network were frozen during training, using weights pre--trained on the ImageNet database. However, the final layer was unfrozen to allow the model to adapt these features for visual field classification.

The model employed the rectified linear unit as the acti-vation function. Thereafter, tegularization was achieved using a 20% dropout technique. Finally, a sigmoid activation function was utilized. The Adam optimizer was selected for its ability to adaptively balance the learning rate during training, and the loss was calculated using the cross-entropy method. The model was configured to train with 40 epochs, and callbacks were implemented to save the best performing model based on the accuracy of the validation set. Given the relative simplicity of the gray-map images, no preprocessing techniques were applied other than standardizing pixel values from 0 to 255 to values between 0 and 1.

A total of 634 low-reliability exams were excluded from the analysis. Of the remaining 885 exams, approximately 70% (n=620) were used for model training, 10% (n=90) were used for validation, and the remaining 20% (n=175) were used for testing. The data were split into one of these sets at the patient level to ensure that the same patient was not included in more than one set. This splitting methodology prevents data leakage and ensures that the model does not learn patient-specific characteristics, thereby improving its generalizability across diverse datasets. Any test result with >20% fixation losses and >15% of false positives, which are the historical and traditional cut-off points used for the SAP-related analysis, were considered low-reliability exams.

The following metrics were used to analyze the model’s performance, considering p<0.05 and 95% confidence interval (CI) using the bootstrapping method:

a) Accuracy: It is a general measure of the AI model’s success. It is calculated from the coefficient between the number of correctly categorized exams and the total number of exams^(10)^.
b) Sensitivity: It refers to the model’s ability to identify truly altered exams. It is obtained through the coefficient between the truly altered exams (true positives) and the sum of the true positive and false negative results. A high sensitivity rate, which indicates a high rate of accurate identification of altered exams^(11)^, is generally desirable in screening tests. The correct identification of altered exams would reduce the number of pathological results that may be missed by the model.
c) Specificity: It refers to the model’s ability to identify normal exam results as negative results. It is calculated by the coefficient of the true negatives and the sum of the true negative and false positive results. High specificity, which indicates a high rate of accurate detection of normal exams^(12)^, prevents an overload of patients with normal exams being referred for further investigation.
d) F1 score: It is the harmonic mean of the accuracy and detection of true positives. It is a balanced measure of the model that is both sensitive and specific. The score varies between 0 and 1, with higher scores indicating better performance^(13)^. F1 score is used when a clear number representing the model’s performance is desirable, especially when dealing with an uneven dataset.
e) Area under the receiver operating characteristic curve (AUC-ROC): It measures the model’s ability to distinguish abnormal results and normal results at diffe-rent classification thresholds^(14)^. A good AUC-ROC is desirable, as the cost of not detecting a false negative results is higher than that of obtaining false positive result. False negative SAPs may delay the treatment of ocular pathologies, increasing treatment costs and impairing prognosis. However, false positive results may be further assessed to strongly establish a diagnosis before initiating treatment.

## RESULTS

**Figure 1:**
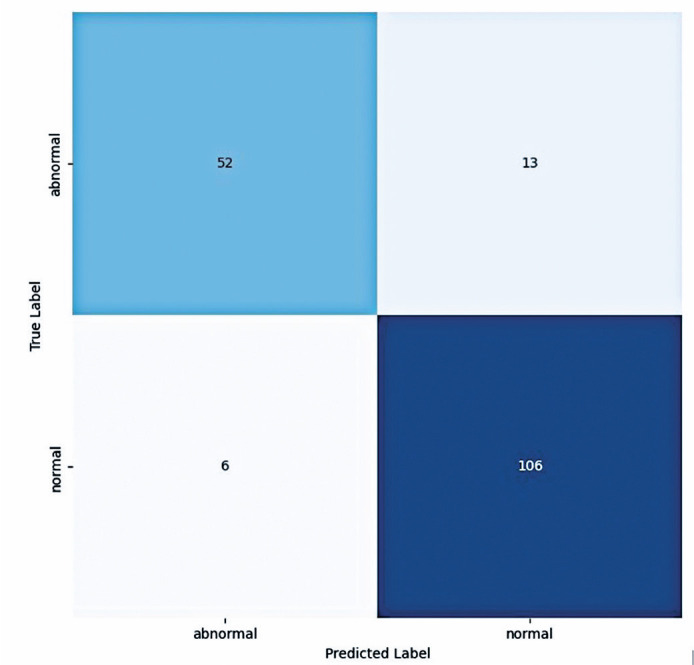
Confusion matrix.

The sensitivity and specificity of the AI model were 80% (68.23%-88.9%) and 94.64% (88.8%-98%), respectively. Using the cross-entropy method, the accuracy and loss rate of the model were 89% and 0.56, respectively (Table 1, Figure 1).

**Table 1:** Statistical analysis of the AI model’s performance

Statistic	Value	95% CI
Sensitivity	80.00%	68.23%-88.90%
Specificity	94.64%	88.70%-98.01%
Accuracy	89.27%	
Loss	56.94%	

* CI= confidence interval.

**Figure 2:**
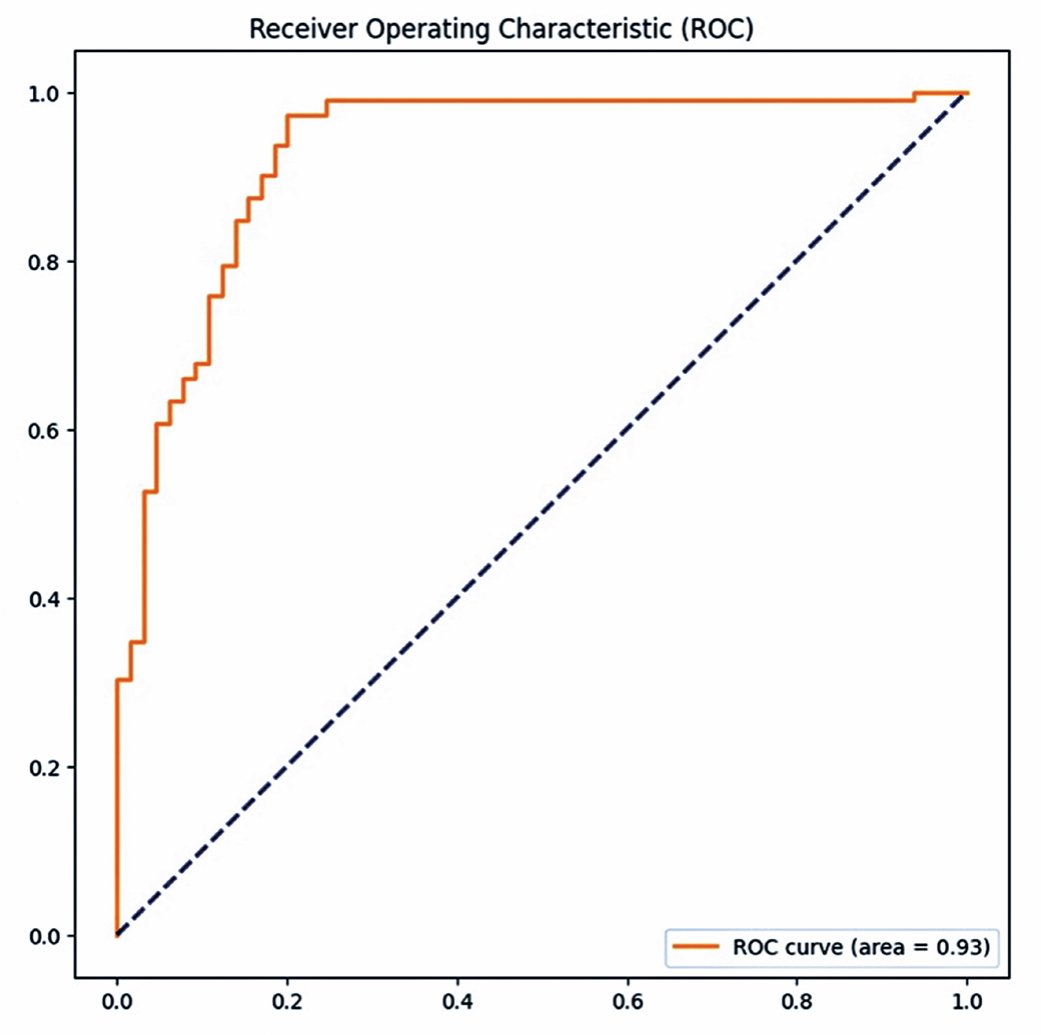
ROC curve of the AI model.

The model achieved an AUC-ROC of 0.93 (Figure 2). In general, an area under the curve (AUC) of 0.5 indicates no discriminatory ability (i.e., ability to differentiate between patients with and without the disease or condition based on the test), AUC of 0.7 to 0.8 indicates acceptable ability, AUC of 0.8 to 0.9 indicates excellent ability, and >0.9 indicates outstanding ability^(15)^.

ROC= receiver operating characteristic; AI= artificial intelligence.

In the test dataset, 65 results were altered and 112 were normal. The model achieved an accuracy of 0.90 and sensitivity of 0.80. The F1 score for the altered and normal exams were 0.85 and 0.92, respectively (Table 2).

**Table 2:** Computational statistics of the AI model’s performance

	Precision	Recall	F1- score	Sample
Altered	0.90	0.80	0.85	65
Normal	0.89	0.95	0.92	112

## DISCUSSION

In recent years, there has been an increase in the consolidation and maturation of technological innovations with the potential to revolutionize different areas of human life, including healthcare. These innovations such as 5G, AI, and ML have created several possibilities and opportunities for healthcare development, which may solve the numerous problems faced by managers and public and private health services. This will ensure universal and sustainable coverage for a rapidly growing and aging population, thereby significantly increasing the demand and cost. Furthermore, these technologies could redefine the way we screen, diagnose, and monitor various pathologies. Thus, in our study, we aimed to present an AI- and ML-based model that is capable of effectively screening for ophthalmological diseases that can alter SAP.

Glaucoma is one of the diseases that alters SAP. It is the main cause of irreversible blindness in the world, affecting approximately 64.3 million people between the ages of 40 and 80. It is estimated that this number will grow to 112 million people by 2040^(16^,^17)^.

It is crucial to have an effective screening method for diseases such as glaucoma because glaucoma is asymptomatic in the early stages, which often causes a delay in diagnosis. Furthermore, by the time the patient seeks medical help for low visual acuity, their glaucoma is in its advanced stages. The cost of treating advanced glaucoma is reportedly four times higher than that of treating early-stage glaucoma. Although most irreversible vision loss can be avoided with early diagnosis and treatment, there are few effective glaucoma screening protocol. This may be attributed to the fact that glaucoma does not meet population screening criteria, with high rates of false positive results when screening individuals with early-stage glaucoma^(18^,^19)^.

There are several ways to apply AI in the diagnosis of ocular pathologies such as glaucoma. Exams such as OCT and color fundus photography have been widely used as a diagnostic and screening method due to their objectivitye. SAP has also yielded interesting results as a screening method^(20^,^21)^.

SAP is a low-cost exam when compared with OCT. Furthermore, any professional or technician is capable of effectively performing the examination without requiring any additional extensive training. However, interpreting the results of SAP is challenging, even by ophthalmologists who are not specialists in the field. This challenge can be mitigated by our AI model which has been trained using reports prepared by experts in the field.

The AI model does not require a professional to interpret the results, thereby reducing the cost of the process and increasing the capacity to absorb the growing demand. Thus, patients with altered exam results can be immediately identified and quickly referred to a specialist for evaluation.

Because our model is based on visual field reports, the application can be used on a simple smartphone with a regular camera, which is available to any physician. This is crucial when dealing with highly prevalent diseases, which require the processing of numerous tests.

Our AI model could also contribute to primary care. SAP could be used for screening at less complex health services, which may aid in prioritizing the referral of patients who need it the most. In areas or cities where there are no ophthalmologists trained to evaluate SAP, the model could partially fulfill the need of an expert, which may help primary care physicians manage the situation properly.

Our model was able to satisfactorily screen and interpret the SAPs, with a sensitivity of 80%. In 1994, a study used a dual-layer neural network to detect glaucomatous changes in visual field examinations, achieving a sensitivity of 65% and specificity of 72%^(22)^. This difference in results may be attributable to the evolution and sophistication of the technology over the years and the fact that the study focused on detecting only glaucomatous changes.

In a 2003 study, neural networks were used to detect the progression of glaucomatous damage, which yielded an AUC of 0.92^(23)^. Another study successfully used neural networks to detect preperimetric glaucomatous damage, achieving an AUC of 0.92^(24)^. Although the purpose of our study was slightly different, the model achieved an AUC of 0.93. Furthermore, the previous study had been performed in an ethnically homogenous Japanese population, whereas our study evaluated a more ethnically diverse population of native South Americans, Southern Europeans, and sub-Saharan Africans.

Our AI model demonstrated an accuracy of 89% in detecting altered perimetry results regardless of the pathology. In one study, visual field defects typical of glaucoma were detected using neural networks with an accuracy of 87%, demonstrating a performance that is superior to other algorithms and even ophthalmologists^(25)^. In another study, a CNN exhibited a strong performance in diagnosing glaucoma, achieving high accuracy in categorizing visual fields as glaucomatous and healthy^(26)^.

AI models have demonstrated advantages in perimetry analysis in terms of process automation, efficiency, and objectivity. When compared with the traditional method of perimetry evaluation by an ophthalmologist, AI models save time and reduce subjectivity in interpreting results. Automating the interpretation of results makes the process faster and more efficient, which reduces the operational costs for managers and health services and enables greater scalability of glaucoma screening programs.

Despite the favorable results, our study has some limitations. The AI model cannot differentiate between the diseases that alter the visual field. Furthermore, the severity of glaucoma may impact the sensitivity and specificity of an AI model. Moreover, different pathologies may yield different results when analyzing them individually. A larger database could improve the model’s accuracy by increasing the data available for training and validation.

In conclusion, we were able to develop an AI model capable of accurately differentiating normal SAPs from pathological SAPs. Thus, it can satisfactorily be used for screening and interpreting SAP results.
